# Towards accessible integrated palliative care: Perspectives of leaders from seven European countries on facilitators, barriers and recommendations for improvement

**DOI:** 10.1108/JICA-03-2017-0006

**Published:** 2017-07-03

**Authors:** Marlieke den Herder-van der Eerden, Benjamin Ewert, Farina Hodiamont, Michaela Hesse, Jeroen Hasselaar, Lukas Radbruch

**Affiliations:** 1 Department of Anaesthesiology, Pain and Palliative Care, Radboud University Medical Center, Nijmegen, The Netherlands; 2 Department of Palliative Medicine, University Hospital Bonn, Bonn, Germany

**Keywords:** Integrated healthcare, Accessibility, Barriers, Facilitators, Quality of healthcare, Integration levels

## Abstract

**Purpose:**

Literature suggests that integrated palliative care (IPC) increases the quality of care for palliative patients at lower costs. However, knowledge on models encompassing all integration levels for successfully implementing IPC is scarce. The purpose of this paper is to describe the experiences of IPC leaders in seven European countries regarding core elements, facilitators and barriers of IPC implementation and provides recommendations for future policy and practice.

**Design/methodology/approach:**

A qualitative interview study was conducted between December 2013 and May 2014. In total, 34 IPC leaders in primary and secondary palliative care or public health in Belgium, Germany, Hungary, Ireland, the Netherlands, Spain and the UK were interviewed. Transcripts were analysed using thematic data analysis.

**Findings:**

IPC implementation efforts involved a multidisciplinary team approach and cross-sectional coordination. Informal professional relationships, basic medical education and general awareness were regarded as facilitators of IPC. Identified barriers included lack of knowledge about when to start palliative care, lack of collaboration and financial structures. Recommendations for improvement included access, patient-centeredness, coordination and cooperation, financing and ICT systems.

**Originality/value:**

Although IPC is becoming more common, action has been uneven at different levels. IPC implementation largely remains provisional and informal due to the lack of standardised treatment pathways, legal frameworks and financial incentives to support multilevel integration. In order to make IPC more accessible, palliative care education as well as legal and financial support within national healthcare systems needs to be enhanced.

## Introduction

Palliative care aims to prevent and alleviate suffering of patients with life-threatening diseases and their families by early identifying and treating their multidimensional symptoms (Sepulveda *et al.*, 2002). Several studies have shown that palliative care is effective in terms of quality of life and costs (Temel *et al.*, 2010; Bakitas *et al.*, 2009; Zimmermann *et al.*, 2014; Dalgaard *et al.*, 2014). However, as problems of accessibility and care fragmentation persist, an integrated care paradigm is increasingly being applied to optimise the quality of palliative care provision (Howlett, 2011; Rocker *et al.*, 2015; Hui *et al.*, 2015; Hui and Bruera, 2015). Integrated care seeks to improve quality of care for patients by ensuring that care is well coordinated around their needs (Goodwin, 2013). It involves various structures and processes that should be pursued at several care levels in order to achieve comprehensive service delivery, addressing individual patient and population needs (Valentijn *et al.*, 2013; Kodner, 2009).

Although integrated palliative care (IPC) lacks a single, commonly agreed definition, a starting point is provided in various sources. For example, the World Health Organization (2016) states among others that palliative care should be provided through person-centred and multidisciplinary care. Furthermore, studies demonstrating the effectiveness of palliative care integration incorporate specific palliative care components (e.g. routine screening, assessment and support of multidimensional symptoms, advance care plans and a multidisciplinary team approach) into standard care for patients with life-threatening diseases (Temel *et al.*, 2010; Bakitas *et al.*, 2009; Zimmermann *et al.*, 2014; Dalgaard *et al.*, 2014). Additionally, several models and indicators to promote IPC are being developed, but these need further evaluation (Hui *et al.*, 2015; Hui and Bruera, 2015). Despite these references to integrated care in a palliative care context, knowledge on models encompassing all integration levels and preconditions for successfully implementing IPC in both oncology and non-oncology is scarce and requires further investigation (Boland *et al.*, 2013; Bruera and Hui, 2012; Gadoud *et al.*, 2013).

The European research project (InSup-C) aims to fill this gap by investigating best and promising IPC practices for patients with cancer, Chronic Obstructive Pulmonary Disease (COPD) and Chronic Heart Failure (CHF) in Europe in order to identify requirements for IPC. In the absence of an IPC definition InSup-C proposed a working definition (Box 1) and published a taxonomy of IPC (Ewert *et al.*, 2016). A predominant part of InSup-C was an international embedded case study examining several promising IPC initiatives (Van Der Eerden *et al.*, 2014). In preparation for this study, qualitative interviews with leaders in the field of IPC were conducted. This paper describes the findings of these interviews, focussing on barriers and facilitators experienced during IPC implementation and recommendations to make IPC more accessible.

## Methods

### Recruitment and sampling

This study used a qualitative interview design which allows for obtaining in-depth insight into how IPC is currently implemented and what factors challenge or promote implementation in InSup-C partner countries. Leaders in the field of IPC in InSup-C partner countries (Belgium, Germany, Hungary, the Netherlands, Spain and the UK) were purposively recruited. Although Ireland was not an InSup-C partner country, one experienced leader that could provide useful insight on IPC implementation in the field of COPD was included. For recruitment each InSup-C consortium member was requested to identify “national leaders” in the realm of IPC. Additionally, letters were sent to boards of national associations of cancer, heart and lung diseases asking for potential leaders in the integration of palliative care in their respective fields. Participants needed to fulfil the following inclusion criteria: knowledge and experience in palliative care and/or public health in cancer and/or non-cancer, working on a minimum degree of local palliative care integration, professional background as physician, nurse, social worker, caregiver, researcher, or patient organisation representative and English communication skills.

**Box 1. InSup-C’s working definition on IPC**

Integrated palliative care involves bringing together administrative, organisational, clinical and service aspects in order to realise continuity of care between all actors involved in the care network of patients receiving palliative care. It aims to achieve quality of life and a well-supported dying process for the patient and the family in collaboration with all the caregivers (paid and unpaid).

A preliminary list included 53 interview candidates. Snowballing during data collection extended the sample to 59 participants. Candidates received an invitation letter explaining the scope of the InSup-C project and the aim of the interview. In total, 34 participants accepted the invitation (eight from the UK, six from Germany, six from the Netherlands, six from Spain, four from Hungary, three from Belgium and one from Ireland; Table I). Two participants accepted the invitation but no date for the interview could be found. In total, 17 participants did not respond to the invitation letter; six participants rejected the invitation. Although we realise that these numbers are not balanced across countries and therefore not sufficient to achieve saturation in terms of regional themes, the interviews provided saturation in terms of shared elements of IPC practices, barriers and facilitators.

### Data collection

Semi-structured interviews were conducted using an interview guideline focussing on the process behind IPC interventions and covering five key dimensions:

1. Definition of IPC.
2. IPC interventions in the participant’s country and beyond based on the IPC working definition.
3. If interventions named: description of the intervention for which the participant could provide most detailed information. If no interventions named: how is palliative care integrated in the work setting?
4. Threats and obstacles with implementation.
5. Characteristics for a successful patient-centred model for IPC.

As patients were not included in the study, approval from a research ethics committee was not required. All experts provided their verbal consent before being interviewed. The interviews were conducted face-to-face (12 interviews) and via Skype (22 interviews) depending on participants’ preferences and logistics. Interview duration averaged 42 minutes. Interviews were audio taped and transcribed verbatim.

### Analysis

**Figure 1:**
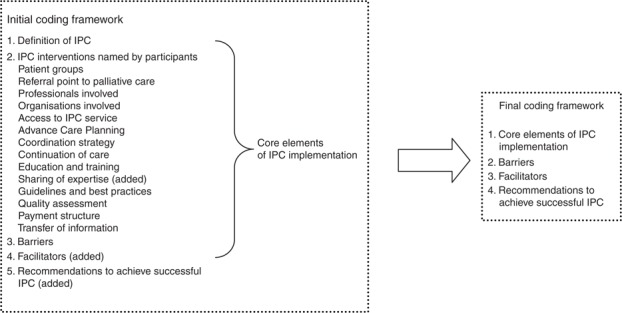
Initial and final coding framework

Transcripts were analysed using thematic data analysis (Marshall and Rossman, 1999) allowing identification of themes in advance while being flexible to add new themes emerging from the interview data. Transcripts were first coded using a coding framework that was based on the interview guideline. Two researchers coded the first five interviews together. The remaining interviews were coded separately. Results were compared and discussed, resulting in three additional codes (“Sharing of expertise”, “Facilitators” and “Recommendations to achieve successful IPC”). As four categories predominated, the final coding framework included four key categories (core elements of IPC practices, barriers, facilitators, recommendations to achieve successful IPC). Figure 1 shows the initial and final coding framework. Quotes were selected in order to illustrate the findings. Data analysis software programme Nvivo 10 was used to support the analysis. The consolidated criteria for reporting qualitative studies (COREQ) checklist (Tong *et al.*, 2007) were used as far as this was applicable to this study.

## Results

Table I presents participant characteristics of which the majority were male. Professional backgrounds were diverse with a majority of physicians with different specialities. The four key categories are described in the following section.

### Core elements of IPC implementation

IPC implementation mainly involved a multidisciplinary team approach including links between hospitals and home care services or sometimes hospices. Palliative care specialists often had an advisory function to support professionals who had basic palliative care training. In some cases palliative care physicians had a more active treating role. At a clinical level a large variety of patient groups was targeted, including patients with malignant and non-malignant diseases (e.g. COPD, CHF, neurologic illnesses). However, at places where IPC was provided for all disease types the majority of patients were affected by cancer. Points of referrals to palliative care differed considerably according to participants. Mechanisms for identification and referral of patients with palliative care needs to palliative care services were not incorporated into standardised treatment pathways, but were rather formed during multidisciplinary meetings (MDTs) where clinical judgment had an important role. Coordination of care was often not standardised in treatment pathways either, although most initiatives made use of professionals fulfilling a key worker role and MDTs to coordinate care. MDTs were not only a means for coordination and communication among professionals, but also had a networking and educational function. Overall, information was mainly transferred through informal communication channels, such as regular phone calls. Many participants used an electronic patient record, but these were often setting-bound or only accessible by the IPC team. Only two participants reported making use of an electronic patient record that was widely accessible across the region or network.

### Facilitators

Facilitators to implementation of IPC focussed on creating awareness about palliative care’s added value and building expertise. In particular, this included the importance of informal professional relationships, basic medical education and general awareness. Above all, participants described how informal relationships between departments and professionals facilitated small-scale multidisciplinary collaborations: “[…] there was a good basis on which we worked together and because it was lots of joint working and lots of cooperation between existing services the oncology team and the lung specialists and this new care service” (UK No. 5; physiotherapist working in outpatient palliative care).

These collaborations, in turn, facilitated dissemination of palliative care expertise: “because we work closely with the local hospice the advanced care practitioner there and the consultant in the hospice they are very good at using the opportunity for feedback also as a learning opportunity” (IR No. 1; nurse working in hospital).

At a professional level, participants found that professionals and particularly non-cancer specialists (e.g. surgeons, pulmonologists, cardiologists) have increasingly accepted palliative care involvement: “I think, you know, in the last ten years I feel shift in that and it is probably likely to continue” (UK No. 3; palliative care consultant working in a hospice). In many of the interviews participants related this growing acceptance to increased basic education in terms of palliative knowledge and skills: “[…] mainly in education. This degree on subspecialty and the other about obligatory trainings for young physicians it’s a big, big result. I am very happy with this and perhaps it’s a step to the integrated palliative care” (HU No. 4; mental-health counsellor working in a hospice).

In addition to growing professional awareness, some participants felt that there was greater recognition of the importance of IPC from key stakeholders, including professional associations and insurance companies. This was resulting in increased interest, new legislation and greater funding for palliative care: “[…] we had in 2002 the law of palliative care with, well funded, government made enough [provided] money for palliative care in Belgium” (BE No. 1; palliative care specialist working in a hospital and teaching at university).

### Barriers

Although IPC is growing in importance, participants experienced a series of barriers when trying to implement IPC in practice. These related to a lack of knowledge about when to start palliative care, lack of collaboration and lack of appropriate financial structures.

Many participants experienced late palliative care integration with inappropriate referrals related to insufficient knowledge about when to start palliative care and healthcare systems’ focus on curative treatment. Despite growing awareness of palliative care, many people felt that it is still often used as a synonym for the care of terminally ill cancer patients, especially in the field of non-cancer: “I mean, healthcare professionals continue with some kind of expectations about palliative care. That means that palliative care patients are patients that are going to die in a short period of time. […] You know, in the last days […] in [the] end of life. And this vision continues” (ES No. 4; palliative care physician working in a hospital). Interviews also revealed that, especially in hospital settings, death is still a taboo, complicating access to palliative care.

Despite the increased willingness to collaborate, participants felt that fragmentation, traditional silo-based ways of working and lack of structures for professional integration (e.g. lack of formalised meetings and non-standardised procedures for information transfer between sectors and team members) made collaboration difficult: “And finally there has been a push for multidisciplinary working for patients with severe heart failure, which is on their mind. [However], I’m not sure that happens well in clinical care, I think conceptually people accept this, but I think putting that into practice in a clinically workable form I think it’s quite difficult” (UK No. 7; cardiologist working in a hospital).

Also at system level, participants met various challenges from financial structures hindering IPC implementation: “Especially, sectorial fragmentation and coexisting financial structures prevent an integration of palliative care into the regular system of service provision” (GER No. 5; oncologist working in a hospital).

Resources were also not always sufficient to ensure IPC access for all patients in need of palliative care. These were not just financial, but could also include lack of skilled staff and lack of time. Lack of finances affected the number of beds in hospices, but also resources needed to enable multidisciplinary care provision: “Our healthcare system is very much affected by the recession. So a lot of our patients could live at home. But there isn’t the social service available for people that don’t have primary carers that are capable of looking after them” (IR No. 1; nurse working in hospital).

### Recommendations for improvement

Participants identified considerable potential to improve IPC implementation in order to make it more accessible. Recommendations included improving IPC accessibility for non-cancer groups, increasing patient-centeredness in care organisation, enhancing multidisciplinary collaboration and providing financial support.

Many participants expressed the wish to upscale IPC implementation to non-cancer patients. A number of them perceived that IPC is often related to diagnosis or prognosis and therefore excludes many (particularly non-cancer) patients. Participants recommended a shift towards needs-based approaches: “They work best and palliative care works best when it is not restricted by a specific diagnosis. So [instead of] the cancer-non-cancer I guess just the progressive illness or advanced progressive illness because that embraces the sort of specialties views of a person-centred care and needs-based intervention it shouldn’t be about someone’s diagnosis specifically” (UK No. 7; cardiologist working in a hospital).

This also required health systems to shift from exclusive end of life care to inclusive and widely accessible palliative care: “[…] we have got to become much more flexible in our models of integrating ourselves with other services and reduce our kind of hospice-centred profile of ‘coming to the hospice and we’ll take care of you until you die’ approach, which is really an unsustainable model, it’s a Rolls Royce service for a few patients, but what we really [got to] get out there is a, is a Mercedes Benz for everybody” (UK No. 8; physician working in a hospice). For some participants, greater accessibility also implied the need for an increased number of beds on palliative care wards and hospices.

At the clinical level, a number of participants identified the need for increased patient-centredness in order to improve care organisation: “I think that the patient has to be in the middle and we all work around him” (ES No. 6; geriatrician working in hospital). Suggestions to enhance patient-centredness included increased patient involvement in the decision process and more patient contact (e.g. through more consultation time and, according to one participant, more bedside consultations).

At a professional level, many participants found that persisting routines of professionals working in isolation with palliative care as only a final stage needed to be broken up. They recommended establishing new routines in which multidisciplinary teamwork and care coordination were standard practices. Following from this recommendation, many participants suggested enhancing palliative care education and some recommended improved guidance on recognition of needs and triggers for referral: “[…] what we also need for integrated palliative care is maybe some more guidelines and […] standardised procedures is maybe a little bit too strong, but some kind of a guiding through how to treat patients in palliative care and I think, well, another thing we need, is, is making physicians more aware of when you start palliative care […]” (BE No. 2; geriatric and palliative care physician working in hospital).

For a number of participants this raised the need to implement IT systems that can improve information exchange and thus enable multidisciplinary care: “We should do a kind of more with telecommunication, with computers, that once a week you make a little meeting with telecommunication, maybe with the patients included, to see if everybody is happy, to see ideas, to ask the question if the palliative care specialist has to come or another caregiver, so maybe that would be a good step” (NL No. 6; cardiologist working in an outpatient clinic at hospital).

Finally, this required financial support at system level: “In my opinion, integrated palliative care, as it is discussed internationally, requires a well-orchestrated cooperation of healthcare institutions and organisations, caregivers and – last but not least – also funding agencies” (GER No. 5; oncologist working in a hospital).

## Discussion

IPC implementation in this study mostly takes place on an informal and small-scale basis. It is mainly realised because of palliative caregivers’ networking capacities, facilitated by policies on raising awareness among physicians and populations which has resulted in increased acceptance and support. However, barriers at clinical, organisational and financial level keep IPC practices at an early stage of development instead of achieving accessible IPC systems at larger scale. Participants recommend that patient-centred, needs-based approaches, integrated (financial and organisational) structures and clinical guidance are required at all healthcare levels to achieve IPC implementation.

Valentijn *et al.* (2013) have developed a conceptual framework useful for understanding the complexity of integrated care. They distinguish integration at several levels that complement each other: system integration at macro level; networking, organisational and professional integration at meso level and clinical integration at micro level. Functional and normative integration link all integration levels together. Valentijn *et al.* (2013) emphasise that integration should take place at all levels across the health system in order to achieve integration and person-centred care. Applying Valentijn *et al.*’s (2013) conceptual framework of integrated care to the study results suggests that access to IPC has developed unevenly at different levels. Most strikingly, the extent of integration achieved in the perception of participants seems to gradually diminish from micro to macro level. IPC has most likely succeeded at the level of clinical integration (micro level) where MDTs stimulate (early) referral of patients with palliative care needs and coordination functions have been employed in order to ensure continuity of care. At the meso level, professional and organisational integration have been achieved to an extent, with professionals establishing informal networks enabling key features of integration such as multidisciplinary treatment which transcends organisational boundaries. However, if such approaches towards integration are not carefully nurtured (or are non-standardised or even non-existent), professionals (e.g. GPs, medical specialists and palliative care specialists) and healthcare providers (e.g. outpatient services, hospice and hospitals) can have difficulties with integration. System integration (macro level) is seen as a key mechanism for upscaling IPC but, so far, is considered mostly incomplete by participants. Many of them found that further IPC implementation not only requires increased education, but also administrative reforms ensuring sustainable financing and organisation of IPC. This study’s findings suggest that current IPC implementation efforts remain rather provisional and fragmented due to the structural shortcomings of different healthcare systems.

Barriers and facilitators described in this study are similar to the existing literature in the field of palliative care in general (Lynch *et al.*, 2009; Von Roenn *et al.*, 2013; Aldridge *et al.*, 2015; Gomez-Batiste *et al.*, 2016), demonstrating the international importance of these themes. However, this study particularly focussed on what barriers and facilitators participants face when actually implementing IPC. The results confirm that in order to promote IPC, integration should take place at several levels of care instead of only adding palliative care as a new specialist silo to standard care at one level. This insight can be used to guide the development of new models of IPC (Ewert *et al.*, 2016), improving existing models of integrated cancer care (Hui *et al.*, 2015; Hui and Bruera, 2015).

### Strengths and limitations

This study’s strength is its international scope with a sample of 34 participants from seven European countries providing rich data. The results highlight that solutions to implementing IPC may not be limited to national boundaries. A major limitation of the study is its small sample reducing its generalisability. For this reason, underlying factors enabling or disabling IPC implementation at a regional level could not be explored in-depth. Although the consistency of our results with other studies supports the validity of the findings, further research with an additional sample of IPC leaders may be helpful to confirm our findings. As a result, another InSup-C study by Centeno *et al.* (2017) investigated leaders’ opinions on barriers and opportunities to the integration of palliative care according to levels of service provision across Europe. Notwithstanding these limitations, this study is a good starting point for further in-depth investigation of IPC. As a next step, a multiple embedded case study with 23 promising IPC initiatives in five European countries was conducted in order to examine IPC implementation in-depth and identify elements for successful IPC integration (Van Der Eerden *et al.*, 2016). A publication of this study is forthcoming.

The predomination of the sample by physicians may have biased our results, meaning that IPC practices with non-medical elements (e.g. integration of palliative care and social care) were possibly underrepresented. Notwithstanding this, the physicians in our sample came from various backgrounds including both oncologists and non-oncologists, allowing us to show that barriers and facilitators towards IPC implementation were not unique for cancer.

## Conclusion

This study suggests that IPC implementation is taking place at several levels of integration. However, IPC implementation efforts remain provisional and informal due to insufficient palliative care knowledge and a lack of standardised treatment pathways, legal frameworks and financial incentives to support multilevel integration. Therefore, the extent to which IPC is realised or not seems to depend too much on professionals’ discretionary engagement and available local resources. In order to manage the leap from local IPC implementation efforts to accessible IPC services at larger scale, education as well as legal and financial support within national healthcare systems need to be enhanced.

## Figures and Tables

**Table I:** Participant characteristics

	*n*
*Gender*	
Male	22
Female	12
*Professional background*	
Physicians	24
Healthcare researcher	3
Nurses	3
Physiotherapists	2
Clinical psychologist	1
Mental-health counsellor	1
*Country of origin*	
UK	8
Germany	6
The Netherlands	6
Spain	6
Hungary	4
Belgium	3
Ireland	1
